# Book Reviews

**Published:** 1912-09

**Authors:** 


### BOOK REVIEWS

PHYSIOLOGY, A Manual for Students and Practitioners. By A. E. Guenther, M. D., and T. C. Guenther, M. D. Second Edition, thoroughly revised and illustrated. Published by Lea & Febiger, Philadelphia.

The authors have gathered within brief compass the facts of physiology with which the medical student should be familiar. The demand which exhausted successive printings of the first edition indicates how well the book has answered its purpose.

A TEXT-BOOK OF PATHOLOGY FOR STUDENTS OF MEDICINE. By J. George Adami, M. A., M. D., F. R. S., and John McRae, M. D. (M. R. C. P. (Lond.) Illustrated with 304 engravings and 11 colored plates. Lea & Febiger, Philadelphia.

This work is an abbreviation of Adami's splendid large volumes upon the same subject. It is simplified in many respects and emphasis placed upon the more important sections so that the student may grasp more readily the essntials. In fact, for the average practitioner, this present simplified volume is more useful than the larger work.

---

THE SURGICAL CLINICS OF JOHN B. MURPHY, M. D., at Mercy Hospital, Chicago. Volume I. Number II. Octavo of 291 pages, illustrated. Philadelphia and London: W. B. Saunders Company, 1912. Published Bi-Monthly. Price per year: Paper, $8.00; Cloth, $12.00.

The second number of this somewhat unusual publication contains facts elaborated from Murphy's famous clinic. expressed in his trenchant style and, one might say, representative of the best surgery done in America.

---

MINOR AND EMERGENCY SURGERY. By Walter T. Dannreuther, M. D., Surgeon to St. Elizabeth's Hospital and to St. Bartholomew's Clinic, New York City. 12mo volume of 226 pages, illustrated. Philadelphia and London: W. B. Saunders Company, 1911. Cloth, $1.25 net.

This book has the distinction of being designed and adapted exclusively to the needs of the hospital interne. It well fulfills its purpose. In fact, the general practitioner, surgeon and specialist may read it with much profit.

---

THE CARE OF SKIN AND HAIR. By William Allen Pusey, A. M., M. D., Professor of Dermatology in the University of Illinois. D. Appleton & Co., New York.

Pusey has written a book chiefly on the hygiene of the skin and hair. The facts should be more generally known by all intelligent persons, and especially by physicians.

THE IMMEDIATE CARE OF THE INJURED. By Albert S. Morrow, M. D., Adjunct Professor of Surgerp in the New York Polyclinic. Second Edition, Revised. Octavo of 354 pages, with 242 illustrations. Philadelphia and London: W. B. Saunders Company, 1912. Cloth, $2.50 net.

This book furnishes a reliable guide for those who wish to learn how to render safe and efficient aid in accidents and other emergencies. The second edition has undergone a thorough revision.

---

TUMORS OF THE JAWS. By Charles L. Scudder, M. D., Surgeon to the Massachusetts General Hospital. Octavo of 391 pages, with 353 illustrations, 6 in colors. Philadelphia and London: W. B. Saunders Company, 1912. Cloth, $6.00, net; half morocco, $7.50, net.

This is truly an age of monographs, and undoubtedly in them we find the best medical literature. Scudder has presented vivid pictures of tumors of the jaws by statistics, case histories, and photographs, so that the physician may recognize new growths of the jaws in their early stages when the proper advice to the patient means so much to him.

